# Mixed reality enhances interobserver reliability in tibial plateau fracture classification compared to computed tomography, 3D computed tomography and 3D printing

**DOI:** 10.1002/jeo2.70474

**Published:** 2025-10-30

**Authors:** Tobias Dust, Julian‐Elias Henneberg, Maximilian J. Hartel, Tobias Malte Ballhause, Christian Arras, André Strahl, Johannes Keller, Anna Streckenbach, Karl‐Heinz Frosch, Matthias Krause

**Affiliations:** ^1^ Department of Trauma and Orthopaedic Surgery University Medical Center Hamburg – Eppendorf Hamburg Germany; ^2^ Department of Trauma Surgery, Orthopaedics and Sports Traumatology BG Hospital Hamburg Hamburg Germany; ^3^ Department of Psychosomatic Medicine and Psychotherapy, Centre for Internal Medicine University Medical Center Hamburg – Eppendorf Hamburg Germany; ^4^ Department of Diagnostic and Interventional Radiology and Nuclear Medicine University Medical Center Hamburg‐Eppendorf Hamburg Germany

**Keywords:** 3D printing, 10‐segment classification, AO classification, diagnostics, mixed reality, revisited Schatzker classification, tibial plateau fracture

## Abstract

**Purpose:**

Tibial plateau fractures present a diagnostic challenge due to complex morphology and demanding anatomy. Reliable diagnosis is essential for treatment planning and clinical outcomes. Prior studies showed benefits of 3D computed tomography (3DCT) and 3D printing (3DP) using CT‐based systems, but the potential of mixed reality (MR) remains unexplored. This study tested the hypothesis that MR enhances spatial understanding of fracture morphology, thereby improving interobserver reliability and diagnostic confidence across modern classification systems compared to CT, 3DCT and 3DP.

**Methods:**

Twenty‐two intra‐articular tibial plateau fractures (Arbeitsgemeinschaft Osteosynthese [AO]/OTA type B or C) were assessed using four visualisation modalities: (1) CT, (2) 3D reconstructions, (3) 3D‐printed models and (4) MR holograms (Microsoft HoloLens 2). Twelve raters (six junior and six senior surgeons) classified each fracture using AO/OTA, 10‐segment and revisited Schatzker systems and rated their diagnostic confidence using a 5‐point Likert scale. Interobserver reliability was measured via kappa coefficients and percentage match. Differences were analysed using one‐way analysis of variance (*p* < 0.05).

**Results:**

MR demonstrated the highest interobserver reliability for all classification systems: AO/OTA (0.43 vs. 0.32 for CT), 10‐segment (0.17 vs. 0.11) and revisited Schatzker (0.43 vs. 0.24). Diagnostic confidence for AO/OTA differed significantly between modalities (*p* = 0.045), with the highest values observed for 3DP (76%) and MR (58%) compared to CT (47%). Revisited Schatzker showed the largest reliability gain, though confidence declined compared to 3DP (59% vs. 34%).

**Conclusion:**

MR improved classification reliability and diagnostic confidence for tibial plateau fractures compared to CT, 3DCT and 3DP. The effect was most evident in less‐experienced surgeons, indicating MR's value for surgical training and preoperative planning. Further research should explore MR's integration into education and its impact on clinical outcomes.

**Level of Evidence:**

Level II.

**Abbreviations**
3DCT: 3D computed tomography
3DP: 3D printing
AO: Arbeitsgemeinschaft Osteosynthese
CT: computed tomography
DICOM: Digital Imaging and Communications in Medicine
FDM: fused deposition modelling
MR: mixed reality
PACS: picture archiving and communication system
PM: percentage match
STL: Standard Tessellation Language
TPF: tibial plateau fracture

## INTRODUCTION

The surgical management of complex tibial plateau fractures (TPFs) continues to be a challenge in orthopaedic surgery. The complexity is compounded by the demanding anatomy of the knee joint and the high variability of fracture patterns, especially in elderly patients [2, 14, 23, 33, 38, 50]. A comprehensive preoperative assessment of the radiographic material, in conjunction with the mechanism of injury and the underlying soft‐tissue trauma, is essential for the determination of the optimal operative strategy [13, 14, 25].

One of the earliest attempts to categorise these fractures was the Schatzker Classification System, which, since its publication in 1979, has provided an early method for classifying fractures into six categories based on two‐dimensional plain radiographs [44]. Despite its widespread use, the Schatzker system often fails to capture the full extent of fracture complexity due to its poor reliability [12]. This is demonstrated by the development of three‐dimensional (3D) technologies, such as computed tomography (CT), which have revealed the limitations of 2D visualisation by incorporating the axial dimension into modern classification systems [12, 20, 24, 32, 40].

Notable among these are the 10‐segment and the revisited Schatzker classification system. These new classifications extend traditional systems by providing a more detailed analysis of fracture patterns, potentially leading to more patient‐specific and algorithmized treatment strategies [20, 24]. Similarly, the Arbeitsgemeinschaft Osteosynthese (AO)/OTA classification system, with its focus on the morphological characteristics of fractures, provides a more robust framework for categorising and managing TPFs [9].

As a subsequent step, the implementation of 3D imaging technologies, including 3D CT scans and 3D printing, has significantly advanced the preoperative planning process by providing a more comprehensive and stereoscopic understanding and enhanced reliability and confidence in the diagnosis of fractures [6, 10, 13, 21, 30]. However, the integration of mixed reality (MR) technology may represent a further breakthrough. MR enables the integration of virtual 3D models into the physical world, offering surgeons an interactive and immersive visualisation tool that could potentially overcome the limitations of existing diagnostic methods [3, 29, 31]. By enabling direct interaction with holographic reconstructions of fractures, MR technology holds the potential to enhance classification accuracy, refine surgical planning and ultimately contribute to improved patient outcomes [27, 28]. However, its diagnostic efficacy in complex TPFs remains uncertain, and a comparison of modern axial fracture classification in terms of reliability testing as the primary endpoint has yet to be conducted.

The purpose of this study is to critically evaluate the impact of MR on the interobserver reliability of the modern classification systems and its use by surgeons with varying levels of experience. It is hypothesised that the selection of a specific CT‐based classification system will influence the diagnosis and therefore operative management strategy of TPFs.

## METHODS

A total of 22 cases of intra‐articular TPFs (AO/OTA type B or C) between 2017 and 2022 were included in this retrospective, diagnostic study (7 women and 15 men, mean age 49 ± standard deviation [SD] 19 years). The fractures were classified according to the AO/OTA system as Type B and Type C. To be eligible for inclusion, each case required a CT scan with an axial slice thickness of 1 mm or less. Twelve raters of varying experience were recruited for the analysis, including six junior and six senior surgeons. Written informed consent was obtained from all participants prior to their inclusion in the study.

### Image acquisition and preparation

CT scans of TPFs were obtained from the institution's picture archiving and communication system (PACS) and stored in Digital Imaging and Communications in Medicine (DICOM) format. Inclusion criteria were a fracture severity of at least B according to the AO classification, no previous fractures of the proximal tibial plateau, and comprehensive preoperative imaging documentation. This documentation had to include digital radiographic anteroposterior and lateral views of the knee, as well as CT scans with a maximum axial slice thickness of 1 mm and multiplanar reconstructions in the sagittal and coronal planes.

Videos were generated from DICOM images (axial, sagittal and coronal sequences of the CT scan) using Horos software. Each video, consisting of 40 images, was integrated into the online survey tool as scrollable frames, emulating the user interface of the PACS system. Similarly, two scrollable 3D reconstructions of the proximal tibia and fibula, with the femur and patella digitally removed, were incorporated into the online survey.

### Segmentation and 3D printing

**Figure 1:**
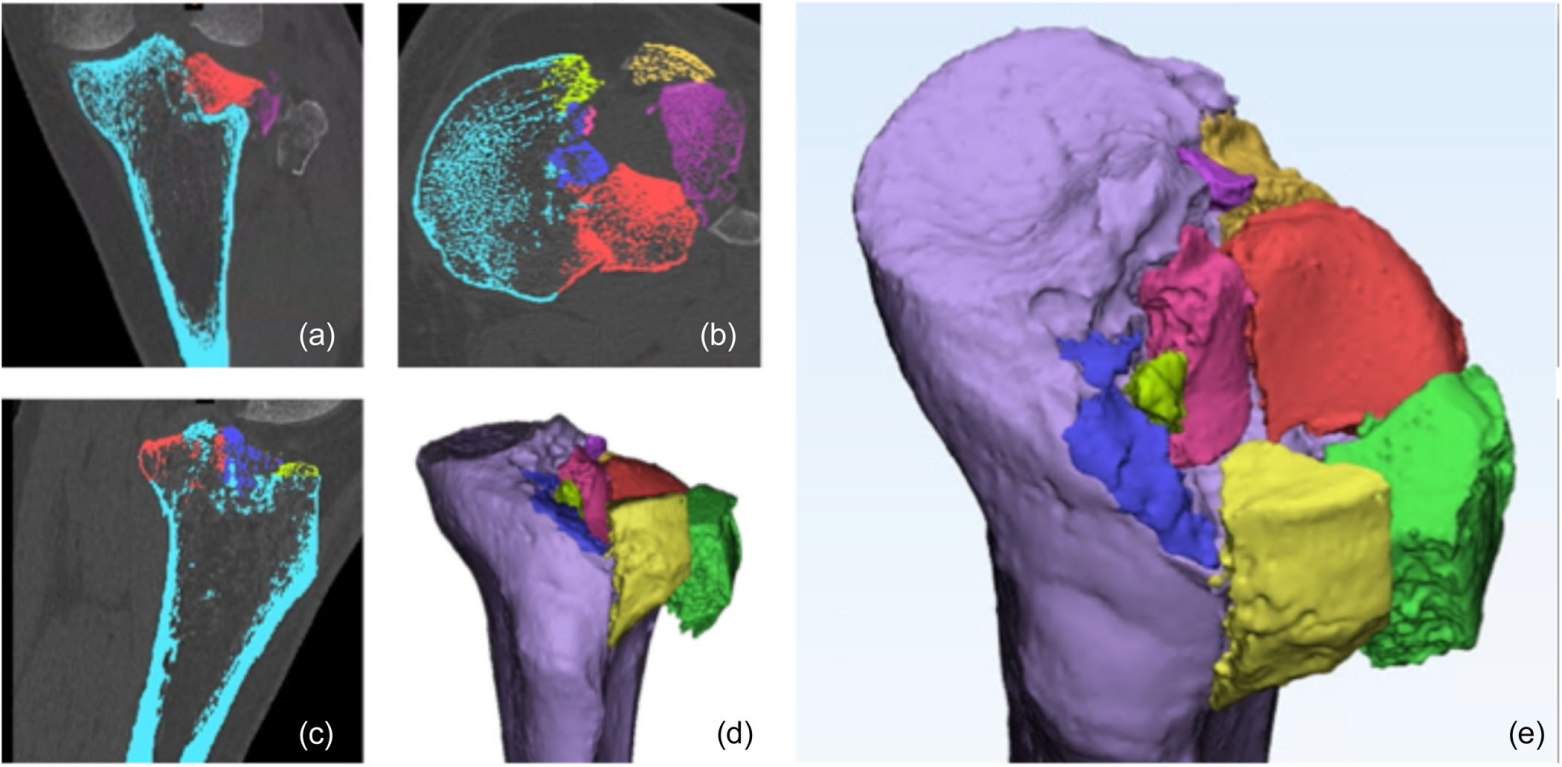
The segmentation process of a unicondylar tibial plateau fracture was conducted using a CT DICOM series (a–c) with the Materialise Interactive Medical Image Control system (Mimics Innovation Suite v24, Materialise, Leuven, Belgium). Following the initial segmentation process conducted with Materialise Mimics (d), the 3D model underwent post‐processing with manual local smoothing and surface fixing using 3‐Matic (e).

CT images of the fractures were archived as complete sets in the DICOM format. These images were then segmented using the Mimics Innovation Suite (Version 24, Materialise, Leuven, Belgium) (Figure 1). 3D models were generated using a semi‐automated segmentation technique that differentiates tissues based on a predefined intensity threshold. Specifically, a threshold of 226 was used to distinguish and separate the bony structures from the soft tissues. The femur and patella were digitally excluded from the field of view to improve clarity of intra‐articular fractures. Due to the presence of impacted and shattered fragments, particularly in complex fracture patterns with a high degree of comminution, manual intervention was required for some segments.

The segmented sections were transferred to the Materialise 3‐Matic software (version 16; Materialise, Leuven, Belgium) for further refinement. This included global surface smoothing and the application of support structures for the larger bone fragments. The final 3D models were then converted to Standard Tessellation Language files for 3D printing and exported as MCS files to the Mimics Viewer (Materialise, Leuven, Belgium) for MR application.

Fractures were printed using an Ultimaker S5 dual‐head fused deposition modelling printer. After printing, the models required the removal of water‐soluble support structures.

### Mixed reality testing

**Figure 2:**
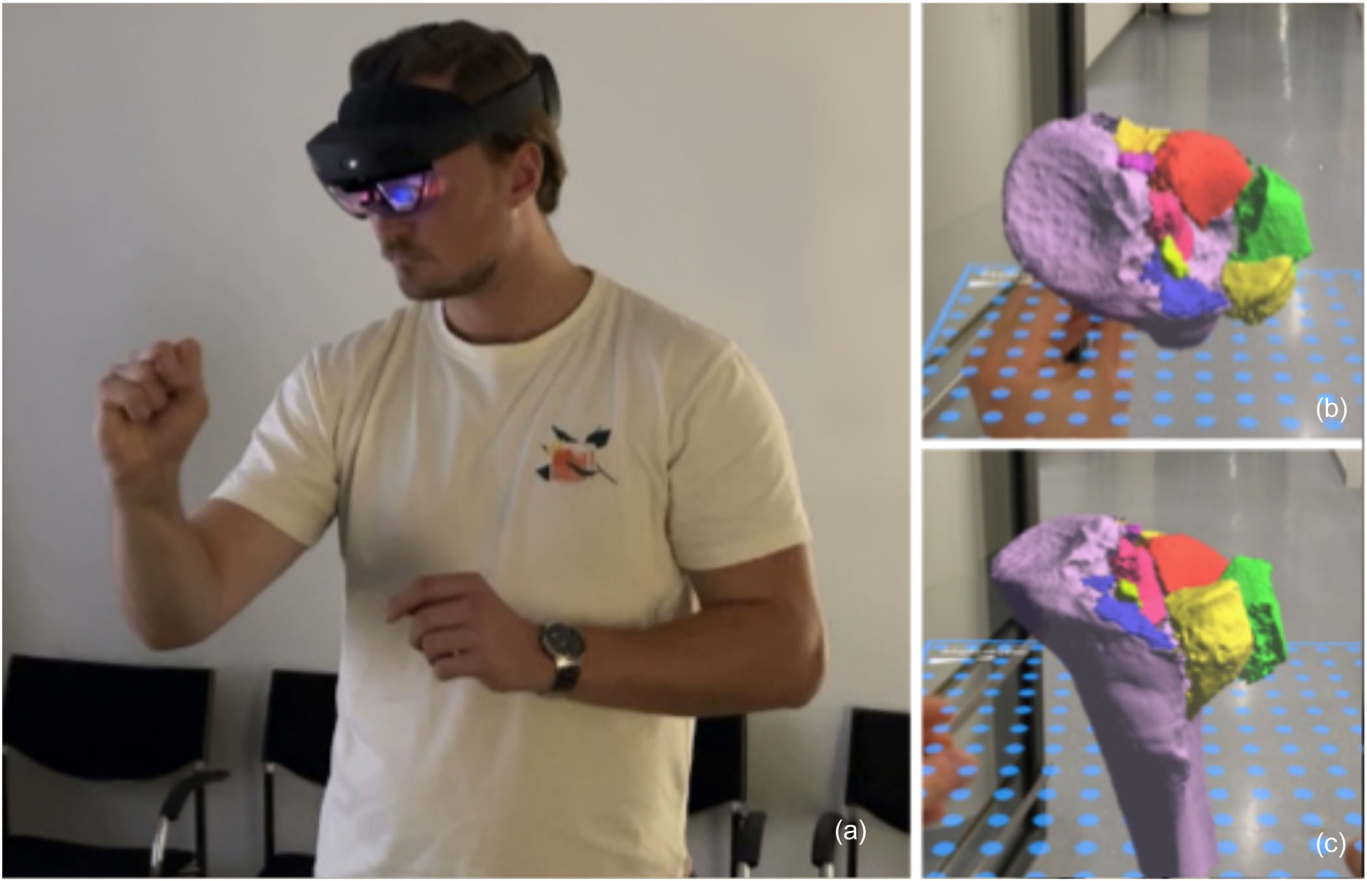
The utilisation of Microsoft Hololens 2 glasses (a) and the visualisation of a multi‐fragmentary tibial plateau fracture in axial and coronal alignment (b and c).

Using Microsoft HoloLens 2, raters were able to interact with 3D reconstructions of fractures in an MR environment (Figure 2). This was done by using the Mimics Viewer application (Materialise, Leuven, Belgium) on HoloLens 2 and uploading the segmented MCS files of all TPFs (Figure 2). The MR content was developed from the same Segmentation datasets used for 3D printing, ensuring consistency across study modalities. Participants were trained to navigate the MR environment prior to assessment.

### Online assessment

Each patient case was systematised into an individual folder using the online platform s2survey.net (SoSci Survey GmbH, Munich, Germany). The folder contained interactive, navigable videos showing the axial, sagittal, and coronal planes of the patient's CT scans, as well as a pair of 3D reconstructions of the tibial fracture. Individual pages within a folder were dedicated to the fracture images from a single imaging modality, accompanied by questions regarding various grading systems and the subjective confidence of the grader. These folders were incorporated into a web page on the survey platform for ease of access.

A total of 12 raters were recruited, each of whom was given an anonymous username and password to access the online platform. These raters were provided with detailed instructions and were blinded to patient cases to prevent bias. To account for raters’ varying levels of experience with common TPF classification systems, summary sheets were distributed that provided a brief overview of these systems. The online survey began with an instructional segment on the classification systems included in the study: the updated AO/OTA classification system, the 10‐segment classification system, and the revisited Schatzker classification system.

**Figure 3:**
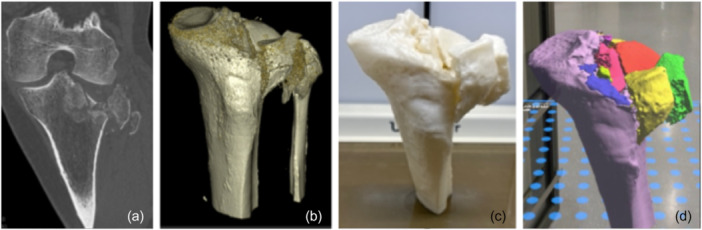
Tibial plateau fracture shown in computed tomography (CT) (a), three‐dimensional (3D) reconstruction (b), 3D printed (c) and mixed reality (MR) reconstructed fracture model (d) imaging modalities.

The survey was structured to be completed in four sequential steps (Figure 3):

First, raters reviewed plain transverse, sagittal and coronal CT images and were asked to classify fracture patterns.

Second, raters used two different 3D reconstructions––one rotatable on the *y*‐axis and the other on the *x*‐axis––to aid in the classification process.

Third, the raters evaluated the physical 3D‐printed models to finalise their classifications and to assess whether the 3D models were useful in classifying TPFs.

Finally, the raters were able to evaluate the TPF through MR glasses and solidify their classifications, also considering the added value of the MR models for classification of TPFs.

At each stage, raters’ confidence levels were measured using a 5‐point Likert scale. The survey was designed to prevent backtracking to previous pages to ensure the validity of the raters’ responses. Questions were presented in a multiple‐choice format with expandable sections for more detailed responses.

Upon completion of the survey, each rater's account was locked to prevent subsequent changes. The study was conducted with the approval of the Institutional Review Board, which ensured that all ethical guidelines were met prior to the start of the research.

#### Primary and secondary outcome

The primary study outcome was to assess the interobserver reliability of TPF classifications across the AO/OTA, 10‐segment, and revisited Schatzker systems. Reliability was quantified using kappa coefficients and percentage match (PM) for each imaging modality (CT, 3DCT, 3D printing and MR). Each fracture was classified by 12 raters (6 junior and 6 senior surgeons), with responses anonymized and blinded to prevent bias.

Secondary outcomes included the diagnostic confidence of raters, which was assessed using a 5‐point Likert scale, as well as the differences in interobserver reliability between junior and senior surgeons across imaging modalities. Additionally, the relative effectiveness of MR in improving fracture classification and diagnostic certainty was evaluated in comparison to CT, 3DCT and 3D printing.

### Statistical analysis

All responses were recorded in an online database, with interobserver reliability calculated using the kappa coefficient and interpreted according to the criteria proposed by Landis and Koch (Table 1) to analyse the reliability for each classification system [26]. In addition, the PM was evaluated as an alternative measure of interobserver reliability [51].

**Table 1:** Landis and Koch grading of reliability based on *κ* coefficient values.

*κ* coefficient	Reliability grading
<0.00	Poor
0.01–0.20	Slight
0.21–0.40	Fair
0.41–0.60	Moderate
0.61–0.80	Substantial
>0.80	Excellent

Five‐point Likert scales were used to measure raters’ confidence in their ratings ranging from ‘I strongly disagree’ (1) to ‘I fully agree’ (5). Data are presented as mean and SD or percentage as appropriate. A univariate one‐way analysis of variance was performed to identify any significant differences between the groups (CT, 3DCT, 3D printing and MR). A significance level of 0.05 was set for statistical analyses, with p‐values below considered statistically significant. SPSS 29.0 (SPSS, Chicago, IL, USA) was used to analyse data using both descriptive and inferential statistical methods.

## RESULTS

### AO classification

The overall reliability of the AO classification demonstrated fair agreement for CT, 3DCT and 3D print, while classification using MR increased its kappa value by 0.10 compared to 3D print, resulting in moderate agreement (CT 0.32; 3DCT 0.32; 3D print 0.33; MR 0.43) according to the criteria proposed by Landis and Koch [26] (Table 2). The overall PM increased from 41% (for CT and 3DCT) to 43% (for 3D print) until 59% (for MR), with the senior surgeons showing the most significant improvement, raising their PM by 30% from 40% (CT only) to 70% (MR) (Table 2). Junior surgeons exhibited a 0.06 increase in kappa values when utilising MR technology compared to CT only, while senior surgeons demonstrated the most substantial improvement, with a 0.28 rise from 0.3 (CT only) to 0.58 (MR).

**Table 2:** Interobserver agreements for the AO classification.

AO classification	CT		3DCT		3DP		MR	
	PM	*κ*	PM	*κ*	PM	*κ*	PM	*κ*
Overall	41%	0.32	41%	0.32	43%	0.33	59%	0.43
Junior surgeons	42%	0.33	41%	0.32	43%	0.34	59%	0.39
Senior surgeons	40%	0.30	42%	0.32	45%	0.36	70%	0.58

Abbreviations: 3DCT, 3D computed tomography; 3DP, 3D‐printing; AO, Arbeitsgemeinschaft Osteosynthese; MR, mixed reality; PM, percentage match.

### 10‐segment classification

The kappa coefficient for the 10‐segment classification demonstrated a general trend of slight agreement, with an improvement observed from 0.11 (CT and 3DCT) to 0.18 (3D print) and 0.17 (MR) (Table 3). The junior surgeons demonstrated the most significant improvement, with a 0.08 increase for 3D print and 0.1 for MR, raising their value from 0.11 for CT to 0.21 for MR. This resulted in a change in their reliability grading from ‘slight’ to ‘fair’. The PM exhibited an 8% improvement, from 71% (CT) to 79% (3D print), with regard to overall agreement when evaluated by CT, 3DCT, 3D print and MR. However, MR demonstrated slightly lower values (76%) in comparison to 3D print (79%). For junior surgeons, the PM exhibited an 8% improvement, rising from 71% (CT) to 79% (3D print) and 7% to 78% (MR). In comparison to the CT‐only evaluation, the senior surgeons demonstrated an improvement of 5% (3D print) and 3% (MR) in their PM values. Notably, the 3DCT exhibited similar PM values to MR (76%).

**Table 3:** Interobserver agreements for the 10‐segment classification.

10‐Segment classification	CT		3DCT		3DP		MR	
	PM	*κ*	PM	*κ*	PM	*κ*	PM	*κ*
Overall	71%	0.11	76%	0.11	79%	0.18	76%	0.17
Junior surgeons	71%	0.11	75%	0.11	79%	0.19	78%	0.21
Senior surgeons	73%	0.13	76%	0.13	78%	0.15	76%	0.13

Abbreviations: 3DCT, 3D computed tomography; 3DP, 3D‐printing; MR, mixed reality; PM, percentage match.

### Revisited Schatzker classification

The revisited Schatzker classification demonstrated overall fair agreement in the kappa coefficient analysis, with the CT‐specific kappa value being 0.24, the 3DCT value being 0.28, and the 3D‐print value being 0.31 (Table 4). The use of MR glasses resulted in an overall agreement of 0.43, indicating a moderate level of agreement. The PM analysis for the revisited Schatzker classification resulted in an overall agreement of 32% (CT), 33% (3DCT) and 35% (3D print), which increased to 58% (MR). Junior surgeons demonstrated an improvement in their PM when using 3D print and MR glasses, with an increase of 3% to 37% (3D print) and 31% to 65% (MR) compared to CT (33%) and 3DCT (34%). The kappa evaluation demonstrated an increase in interobserver agreement kappa values by 0.05 (3D print) in comparison to 3DCT. Additionally, the junior surgeons exhibited a kappa class switch from fair to moderate agreement when utilising MR glasses, with the value increasing by 0.17 to 0.5 in comparison to 3D print. In contrast, the senior surgeons demonstrated a reduction in their kappa values, from 0.25 (CT) and 0.27 (3DCT) to 0.22 (3D print). This trend was also observed in the PM evaluation, with 31% in CT only, 32% in 3DCT and 28% using 3D print. In contrast, the interobserver reliability kappa values increased by 0.32 to 0.54 (MR) when the MR glasses were used, indicating a kappa class switch from fair to moderate. The same results were observed in the PM evaluation, with the MR glasses showing an increase of 36% to 64% compared to the 3D‐print evaluation.

**Table 4:** Interobserver agreements for the revisited Schatzker classification.

Revisited Schatzker	CT		3DCT		3DP		MR	
	PM	*κ*	PM	*κ*	PM	*κ*	PM	*κ*
Overall	32%	0.24	33%	0.28	35%	0.31	58%	0.43
Junior surgeons	33%	0.26	34%	0.28	37%	0.33	65%	0.50
Senior surgeons	31%	0.25	32%	0.27	28%	0.22	64%	0.54

Abbreviations: 3DCT, 3D computed tomography; 3DP, 3D‐printing; MR, mixed reality; PM, percentage match.

### Subjective certainty

The subjective certainty of diagnosis, as indicated by raters selecting the ‘certain’ or ‘very certain’ categories, demonstrated improvement in both the AO/OTA and 10‐segment Classification when 3D printing and MR were utilised (Table 5), with a significant improvement using AO/OTA classification (*p* = 0.045). It is noteworthy that for both the AO/OTA and 10‐segment classifications, there was a notable increase in certainty, ranging from 63% to 92% (10‐segment) and from 47% to 76% (AO/OTA), respectively, when the evaluations were conducted with 3D‐printed models as opposed to CT or 3DCT alone. The utilisation of MR has been demonstrated to result in a reduction in subjective certainty of 18% when AO/OTA classification is evaluated, and a decrease of 21% (from 92% to 71%) has been observed when 10‐segment classification is assessed. Upon evaluation of the revisited Schatzker classification, it became evident that it exhibited the lowest subjective certainty values in both CT and 3D CT when compared to the AO and 10‐segment classification. However, when 3D printing was employed, the raters reported an increase in subjective safety by 21% (CT) and 19% (3D CT) to 59%, which also corresponded to the lowest value observed across all three classification systems. Nevertheless, the utilisation of MR demonstrated a 25% decline in perceived subjective safety, reaching a value of 34%. This is also the lowest observed value across all classification systems.

**Table 5:** Subjective certainty of the rater regarding his decision.

Category: Agree and fully agree	CT	3DCT	3DP	MR	*p*‐value
AO classification	47%	50%	76%	58%	**0.045**
10‐segment classification	63%	65%	92%	71%	0.100
Revisited Schatzker classification	38%	40%	59%	34%	0.253

Abbreviations: 3DCT, 3D computed tomography; 3DP, 3D‐printing; AO, Arbeitsgemeinschaft Osteosynthese; MR, mixed reality.

## DISCUSSION

The accurate classification of TPFs remains a significant challenge for orthopaedic surgeons, largely due to the complexity of these injuries and the well‐known limitations of conventional imaging techniques [8, 12, 13]. Postoperative malreductions are observed in 32,3% of cases, particularly in the posterolateral region of the tibial plateau, which can result in pseudoinstabilities and secondary arthrosis [35, 48]. MR technology may offer a valuable supplementary approach to traditional methods, such as CT and 3D printing, by providing an interactive and immersive visualisation of fractures. This study sought to determine whether MR improves interobserver reliability and diagnostic confidence in TPF classification compared to CT, 3DCT, and 3D printing, using the AO/OTA, 10‐segment, and revisited Schatzker systems with surgeons of different levels of experience. We found that MR improved interobserver reliability among all classification systems compared to CT and 3DCT, benefiting both junior and senior surgeons. However, only the 10‐segment classification systems demonstrate a notable – yet not significant ‐ improvement in diagnostic confidence when utilising the MR environment. MR offers promising potential as a tool for preoperative planning and surgical training by providing an immersive environment for understanding complex fractures and guiding decision‐making.

### Interobserver reliability of tibial plateau fracture classification using MR, CT, 3DCT and 3D printing

The MR environment demonstrated superior interobserver reliability compared to CT, 3DCT and 3D printing across all classification systems. Notably, both the AO/OTA and the revised Schatzker classification system demonstrated superior performance when utilising the MR environment among both junior and senior surgeons. The 10‐segment classification system showed comparable outcomes to those observed in the 3D‐printing group. Several studies have demonstrated the superiority of a 3D‐printed fracture model over CT imaging alone for the diagnosis of complex fractures [1, 4, 13, 16, 39, 45]. However, only a limited number of studies have additionally examined the potential applications of MR in the context of fracture classification [3, 6, 37]. Bitschi et al. were the first to assess the pre‐surgical utilisation of MR technology in the diagnosis of tibial head fractures, although the study was conducted on a limited sample of three fracture specimens [3]. Despite the absence of an interobserver analysis, the 10‐segment classification revealed that MR visualisation resulted in a modification of the selected segments in 79% of cases and in 7% of the Schatzker classification. These findings suggest that the presurgical implementation of an MR environment may provide enhanced benefits when compared to the utilisation of conventional imaging and 3D‐printing techniques. In contrast, Brouwers et al. found inferior reliability results for their 3D‐virtual reality environment compared to 3D printing in acetabular fractures [6].

The inconsistency in results across these studies may be due to the lack of standardisation in the MR environment. In addition to the use of varying hardware (such as virtual reality or augmented reality glasses), various software solutions appear to have a notable impact on usability and quality, which limits the ability to compare results.

### Diagnostic confidence among surgeons of varying experience levels across imaging modalities

The results of this study revealed mixed findings regarding subjective diagnostic confidence when MR was compared to CT, 3DCT and 3D printing, with notable variations across the three classification systems. While 3D printing consistently improved diagnostic certainty across all systems, MR environment showed varying results across all three classification systems.

The raters reported an increase in perceived safety from 63% (CT) and 65% (3DCT) to 71% when employing the MR environment for the 10‐segment classification, although this remained below the 92% achieved with 3D printing. However, subjective safety for the AO/OTA classification was reduced in the MR environment compared to the 3D‐printed models, although it remained higher than in the CT/3DCT environment. Conversely, when utilising the MR environment for the revisited Schatzker classification, the raters demonstrated the lowest perceived safety values among all modalities. Nevertheless, across all classification systems (10‐segment, AO/OTA and revisited Schatzker), the MR environment yielded higher or comparably high interobserver reliability results, despite the differential patterns observed in subjective certainty.

While the MR environment appears to present a notable improvement in fracture imaging [3, 6, 11, 37, 47], the current literature shows no clear enhancement in surgeons’ confidence in presurgical planning. While MR visualisation has shown advantages over CT and 3D printing in improving fracture understanding and treatment strategies for complex TPFs [3, 11], for acetabular fractures, MR demonstrated comparable diagnostic accuracy to CT among orthopaedic residents, while 3D printing offered higher subjective safety values [6, 37]. However, a direct comparison of the influence of the MR environment on the subjectively perceived safety compared to CT and 3D printing remains to be conducted.

In the context of our results, the lower clinical application of the revised Schatzker classification in comparison to the 10‐segment and AO/OTA classifications appears to provide a rationale for the consistently lower confidence values.

### Consistency of classification across AO/OTA, 10‐segment and revisited Schatzker systems using different visualisation techniques

This study demonstrated that the diagnostic added value of the MR environment for TPFs is influenced by the classification system applied. While the AO/OTA and the revisited Schatzker classification systems appear to yield a quantifiable diagnostic added value through the MR environment, even in comparison to 3D printing, the reliability of the 10‐segment classification could not be enhanced by an immersive interface. Several authors have proposed that interobserver reliability may be influenced by the varying levels of clinical experience [7, 42, 46]. However, this effect was not quantifiable in our study. One potential explanation for this phenomenon is the increasing integration of 3D printing and MR in clinical practice, which may be leading to a more widespread familiarity with these techniques among surgeons, regardless of their seniority.

For the AO classification, an increase in interobserver agreement was observed across all levels of experience, with senior surgeons showing the greatest improvement using 3D print and MR environment. The detailed pictograms provided by the AO classification system likely aided the raters in their decision‐making process [34]. Another critical factor was the level of detail required of the raters, as they were asked to select from subgroups using qualifiers, which further contributed to their decision accuracy. The introduction of the MR environment showed an improvement in interobserver reliability, particularly among senior surgeons, with a percentage agreement of 70% and a kappa value of 0.58, outperforming traditional imaging tools such as CT and 3DCT. In comparison, the 3D‐printing group also showed improvement, but with a slightly lower overall agreement (PM = 45% and *κ* = 0.36) proving similar results of earlier studies [3, 6, 13, 19], suggesting that the MR environment may provide a more intuitive and immersive experience, improving fracture interpretation, especially in more complex cases [3, 11, 34].

The interobserver agreement of the 10‐segment classification, assessed by the kappa coefficient, showed certain limitations due to the high number of items in the system. Agreement in nine out of ten selected segments is statistically interpreted as disagreement, resulting in low kappa values [51]. Nevertheless, the PM analysis showed the highest agreement in the overall 3D‐printing group (79%), while the overall kappa values remained low (*κ *= 0.18). The MR environment, however, showed comparable results in terms of percentage agreement (76%) but provided more consistent kappa values, especially among junior surgeons (*κ* = 0.21), suggesting that MR may provide an advantage by offering enhanced spatial visualisation, potentially compensating for the weaknesses inherent in the complexity of the 10‐segment classification [30]. In regard to a reliable diagnosis of complex tibial head fractures, the 10‐segment classification demonstrates a consistently high PM evaluation across all levels of raters’ experience, indicating a significantly higher clinical benefit compared to the AO/OTA and revisited Schatzker classification.

Previous studies have primarily analysed the original Schatzker classification for overall concordance [43]. In 2018, Kfuri et al. revised this classification by introducing a virtual equator dividing the tibial plateau into medial, lateral, anterior, and posterior aspects [20]. Analysis of the revisited Schatzker classification in this study showed an increase in agreement among less experienced graders, while the more experienced graders had lower agreement scores but improved intraobserver reliability. A comparison of the 3D printing and MR environments revealed that the MR system exhibited a higher degree of overall percentage agreement (58%) and kappa value (*κ *= 0.43) than the 3D‐printing group (35% and *κ* = 0.31). Additionally, there was a noteworthy improvement in kappa class from ‘fair’ to ‘moderate’. Notably, junior surgeons particularly benefited from the MR environment, achieving a percentage agreement of 65% and a kappa value of 0.50, highlighting the potential of MR to improve diagnostic accuracy and decision‐making confidence among less experienced clinicians. It is noteworthy that senior surgeons exhibited a slight decline in interobserver reliability when utilising 3D printing in comparison to CT and 3DCT. However, the MR environment demonstrated an increase in both the kappa value and the PM evaluation, with a notable kappa class shift from ‘fair’ to ‘moderate’.

Although both the revisited Schatzker and 10‐segment classifications allow for accurate localisation of fracture fragments, they fall short in adequately representing fracture morphology and displacement (e.g., comminution or depression) [36]. The AO classification, on the other hand, better captures these morphological aspects, but with less precision in fracture localisation [18]. Importantly, the MR environment demonstrated an enhanced ability to visualise both fracture location and morphology compared to the 3D‐printed models, which were limited by their static nature. The immersive nature of MR allowed for dynamic interaction with the fracture model, providing a superior tool for understanding fracture complexity and guiding preoperative planning [3].

#### Mixed reality environemnt

The use of MR in fracture assessment and preoperative planning offers several advantages over conventional and 3D methods. The majority of existing studies regarding interobserver agreement of classification systems of the tibial plateau have primarily focused on conventional imaging techniques, such as plain radiographs, CT scans, 3D volumetric reconstructions and 3D‐printing technology. This study introduces the novel MR environment for preoperative diagnostics, which has been successfully employed as a diagnostic tool in smaller sample sizes and as a precise complement for correct intraoperative imaging in earlier studies [3, 17].

A further crucial aspect in the comparison of 3D printing and MR in fracture diagnostics is the distinction between the processes involved in the production of a 3D‐printed and an MR model. While the technical requirements of the CT image qualities and the segmentation process are largely similar for both models [5], the 3D‐printing models require an average production time of between one and one and a half days. In contrast, the MR preparation in this study was completed directly after segmentation process, which gave the MR environment an advantage in terms of preparation time. It is also important to note that personnel with specialised training are required to ensure the consistent quality of the data preparation and printing process. However, in the clinical practice of the authors, TPFs are not routinely treated immediately but around the fifth day after trauma [15, 41].

### Limitations

This study had a number of limitations. First, it is important to acknowledge the variability in CT scan quality. In order to comply with the radiation safety protocols and ethical considerations, patients transferred from smaller medical facilities were not required to undergo a second CT scan at the primary research centre for research purposes. Only those external CT datasets that satisfied the required technical specifications for 3D printing were included. This approach may have introduced variability in the segmentation process, which could potentially affect the quality of the 3D‐printed and MR models. However, the use of standardised segmentation protocols aimed to minimise these inconsistencies.

Second, the potential influence of fatigue on graders must be taken into account. As previously observed by Wainwright et al., the evaluation of a substantial number of radiographic images over an extended period may result in a reduction in the ability to recognise and differentiate between details [49]. To address this potential bias, the graders were allowed to take scheduled breaks and resume their evaluations at a later point in time. Nevertheless, the possibility of data integrity issues resulting from fatigue cannot be completely eliminated.

Third, the training provided to raters for navigating the MR environment and classifying fractures represents another limitation. Variability in the quality of training may have resulted in differences in familiarity with the technology, thereby introducing variability in scoring. A learning curve inherent to MR technology may have further influenced initial classifications, particularly among raters with less experience with advanced imaging tools. All participants received a standardised 10‐minute introduction covering headset operation, navigation, manipulation of fracture models and interaction with the MR interface. No separate trial or familiarisation set of cases was provided prior to the formal evaluation, which may have limited the opportunity to adapt to the interface and contributed to lower subjective certainty in some classification systems. To reduce this bias, fractures were presented in a random order to all raters.

Fourth, CT‐based axial classification systems are considerably more intricate than 2D‐based systems, offering a wider range of options for fracture description. It has been demonstrated that the disagreement among raters increases, particularly for complex fracture patterns [7, 9, 18, 36]. In addition to the increased complexity, the survey was designed to yield lower kappa values by including a total of 12 raters, as the probability of higher agreement decreases with an enlargement of the number of raters evaluating the fracture. However, the inclusion of numerous raters ensured a substantial degree of redundancy for the measured outcomes. The variability in outcomes across comparable studies may be attributed to differences in the number of cases, the number of raters, varying levels of pretest teaching, and advancements in imaging methods that enhance the likelihood of agreement [36]. To enhance the comparability between the classification systems and the CT/3D‐printing group, an additional reliability analysis was conducted using the PM agreement.

Fifth, in line with the recommendations by Koo and Li, a minimum of 30 heterogeneous subjects is generally advised to ensure stable estimates in interrater reliability analyses [22]. Our study included 22 fracture cases, which—while comparable to or exceeding prior studies —remains below this suggested threshold. Although the relatively high number of raters (*n* = 12) increases robustness, the limited case number may still reduce statistical power and widen confidence intervals. For the analysis of subjective confidence (Table 5), junior and senior surgeons were pooled to maintain statistical stability and to focus on modality‐related trends rather than experience‐level differences, as subgrouping would have further reduced the number of observations per cell and increased variability.

Finally, the lack of standardisation in the clinical application of extended reality technologies (including virtual reality, augmented reality and MR) presents a challenge to the comparability of studies. Significant discrepancies in software and hardware capabilities, such as the restricted model detail supported by the Microsoft HoloLens 2, present a challenge to direct comparisons between studies. Further research is required to investigate the technical variations between extended reality devices and their influence on fracture visualisation. Despite these limitations, the study's findings remain robust. The utilisation of multiple imaging modalities, standardised workflows, and a diverse group of raters provides confidence in the observed improvements in diagnostic reliability and certainty.

## CONCLUSION

The present study demonstrates that the utilisation of MR and 3D‐printing technologies notably enhances the diagnostic accuracy of TPF classification in comparison to traditional imaging methods. Specifically, MR was found to enhance interobserver agreement more effectively, indicating its potential as an advanced tool for fracture evaluation. The AO/OTA classification demonstrated enhanced reliability with the use of MR, while the revisited Schatzker classification exhibited notable benefits for junior surgeons, underscoring the capacity of MR to facilitate the work of less‐experienced clinicians. Furthermore, the 10‐segment classification system demonstrated consistent high overall agreement across all experience levels, emphasising its clinical usability.

The findings indicate that 3D‐printed models are an effective method for enhancing confidence in diagnosis across all experience levels. Moreover, the additional interactive elements offered by MR technology further enhance diagnostic accuracy, particularly in cases of complex fractures. However, the subjective certainty in MR evaluations was occasionally lower, which is likely attributable to the raters’ lack of familiarity with the technology.

Further investigation is required to standardise MR techniques and more effectively integrate this technology into clinical practice, with the objective of establishing its routine use in orthopaedic surgery to achieve optimal patient outcomes.

## AUTHOR CONTRIBUTIONS

All authors drafted the article. Tobias Dust, Julian‐Elias Henneberg, Christian Arras and Tobias Malte Ballhause were responsible for patient selection and 3D‐printing preparation. Tobias Dust, Julian‐Elias Henneberg, Matthias Krause and Maximilian J. Hartel designed the questionnaire and online survey tool. Tobias Dust, Julian‐Elias Henneberg and Anna Streckenbach performed data collection and statistics. Tobias Dust, Julian‐Elias Henneberg and Anna Streckenbach designed the figures. Matthias Krause, Karl‐Heinz Frosch and Johannes Keller revised the article. Karl‐Heinz Frosch, Matthias Krause and Anna Streckenbach provided the theoretical background. All authors read and approved the final version of the article.

## CONFLICT OF INTEREST STATEMENT

The authors declare no conflict of interest.

## ETHICS STATEMENT

The study was approved by the Ethics Committee of the Medical Chamber of Hamburg, Germany (ID 2024‐101279‐BO‐ff) and conducted according to the guidelines of Good Clinical Practice and the recommendations of the Declaration of Helsinki. Written informed consent was obtained from each participant who agreed to be included in the study.

## Data Availability

The data sets generated during and/or analysed during the current study are not publicly available due to privacy and confidentiality concerns related to patient and observer data which are protected under health information privacy laws. The data can be obtained by contacting the corresponding author.
